# *NOTCH1* mutation and its prognostic significance in Chinese chronic lymphocytic leukemia: a retrospective study of 317 cases

**DOI:** 10.1002/cam4.1396

**Published:** 2018-03-23

**Authors:** Yixin Zou, Lei Fan, Yi Xia, Yi Miao, Wei Wu, Lei Cao, Jiazhu Wu, Huayuan Zhu, Chun Qiao, Li Wang, Wei Xu, Jianyong Li

**Affiliations:** ^1^ Department of Hematology the First Affiliated Hospital of Nanjing Medical University Jiangsu Province Hospital Nanjing 210029 China; ^2^ Key Laboratory of Hematology of Nanjing Medical University Nanjing 210029 China; ^3^ Collaborative Innovation Center for Cancer Personalized Medicine Nanjing 210029 China

**Keywords:** Chronic lymphocytic leukemia, cytogenetics, molecular biology, *NOTCH1* mutations, prognosis

## Abstract

The proto‐oncogene *NOTCH1* is frequently mutated in around 10% of patients with chronic lymphocytic leukemia (CLL). This study analyzed *NOTCH1* mutation status of 317 Chinese patients with CLL by Sanger sequencing. The frequencies of *NOTCH1* mutation in the PEST (proline (P), glutamic acid (E), serine (S), threonine (T)‐rich protein sequence) domain and the 3′ untranslated regions (UTR) were 8.2% and 0.9%, with the most frequent mutation being c.7541_7542delCT and c.*371A>G, respectively. Clinical and biological associations were determined including *NOTCH1* mutations with advanced stage (Binet stage, *P *= 0.010), unmutated immunoglobulin heavy‐chain variable region (*IGHV*) gene (*P *< 0.001) and trisomy 12 (+12) (*P *= 0.014). *NOTCH1‐*mutated patients had lower CD20 expression intensity than *NOTCH1‐*unmutated patients (*P *= 0.029). In addition, *NOTCH1*‐mutated patients had shorter overall survival (OS) (*P *= 0.002) and treatment‐free survival (TFS) (*P *= 0.002) than *NOTCH1*‐unmutated patients, especially for patients with *NOTCH1* c.7541_7542delCT and/or c.*371A>G mutations. Patients with both mutated *NOTCH1* and unmutated *IGHV* had shorter OS (*P *< 0.001) and TFS (*P *< 0.001) than those with unmutated *NOTCH1* or mutated *IGHV*. These data provide a comprehensive view of the clinical relevance and prognostic impact of *NOTCH1* mutations on Chinese patients with CLL.

## Introduction

Chronic lymphocytic leukemia (CLL) is a common type of B‐cell chronic lymphoproliferative disorders in adults 1, 2. Some common molecular and cytogenetic abnormities in CLL, such as tumor protein 53 (*TP53*) mutation, *TP53* deletion, immunoglobulin heavy‐chain variable region (*IGHV*) mutations, and *ATM* deletion, are associated with clinical features and prognosis of patients with CLL 3, 4, 5, 6, 7, 8.

*NOTCH1* mutations are frequent in CLL 9. Encoding domains mutations, mainly in PEST (proline (P), glutamic acid (E), serine (S), threonine (T)‐rich protein sequence) domain 10, 11, 12, are reported to occur in nearly 10% patients with CLL in Western countries 3, 13, 14. The mutations increase with disease progression and drug resistance. In recent years, with the application of next‐generation sequencing technology, a better understanding of *NOTCH1* mutations was built 15. Puente et al.^16^ firstly reported noncoding mutations in 3′ untranslated regions (UTR), including c.*378A>G, c.*371A>G and c.*380A>C, with total mutations rate about 5%. Soon afterward, Larrayoz et al. 17 showed that *NOTCH1* noncoding domains mutations occurred in 2.2% of patients with CLL who participated the UK CLL4 trial.

In Western countries, *NOTCH1* mutations have a significant association with unmutated *IGHV* 18. Pozzo et al. 19 found that *NOTCH1* mutations suppressed CD20 expression intensity of B‐cell surface by dysregulating histone deacetylase (HDAC)‐mediated epigenetic changes. As a consequence, rituximab seems to have less benefits to patients with *NOTCH1* mutations than those without mutation regardless of mutation sites 14, 20. Furthermore, *NOTCH1* mutations are associated with trisomy 12 (+12), with nearly 40% mutated patients harboring +12 21, 22, 23. It has been reported that *NOTCH1* abnormality represented a poor prognostic factor in CLL on both treatment‐free survival (TFS) and overall survival (OS) 5, 16, 17. The effects of different types of *NOTCH1* mutations on prognosis were reported by D'Agaro et al. 24. Although Xia et al. 25 and Wu et al. 26 partly investigated *NOTCH1* mutations in Chinese with CLL, so far, there has been no systemic study. It remains unclear that whether *NOTCH1* mutations have a similar influence on Chinese patients with CLL as Western countries.

In this retrospective study, we analyzed correlations between *NOTCH1* mutations and other clinical and prognostic parameters in 317 Chinese patients with CLL. Besides, survival analysis was performed based on *NOTCH1* and other cytogenetic status.

## Materials and Methods

### Patients

This single‐center retrospective study included 317 patients with CLL diagnosed from November 1991 to December 2016 in our hospital. Diagnosis of CLL was based on the International Workshop on CLL‐National Cancer Institute criteria. This study was approved by the hospital ethics committee, and all patients were provided informed consent according to the Declaration of Helsinki. Among 317 patients, mutation status of PEST domain of 161 patients had been reported by Xia et al. 25.

### *NOTCH1* mutation detection

Genomic DNA from CLL samples was extracted as previous report 25. Briefly, PCR amplification was performed for *NOTCH1* PEST domain forward primer 5′‐CAGATGCAGCAGCAGAACCTG‐3′ and reverse primer 5′‐AAAGGAAGCCGGGGTCTCGT‐3′ and 3′ UTR forward primer 5′‐CCTAACAGGCAGGTGATGCT‐3′ and reverse primer 5′‐ATCTGGCCCCAGGTAGA AAC‐3′. Sanger sequencing was performed in PEST domain and 3′ UTR. Variant allelic fraction threshold to detect mutant alleles by Sanger sequencing was 12% according to a previous study 5.

### Cytogenetics and immunophenotyping

Karyotype analysis of CLL cells was performed after CpG‐oligodeoxynucleotide and interleukin‐2 stimulation. Fluorescence in situ hybridization (FISH) was carried out according to the procedures described previously 25. CD38 and ZAP‐70 were detected via flow cytometry, and the cutoff levels for positivity were 30% and 20%, respectively.

### Statistical analyses

SPSS 23 was used to analyze data. OS was defined as time from diagnosis to death or last follow‐up, and TFS was calculated as time between diagnosis and first‐line treatment. Survival curves were constructed by Kaplan–Meier method, and log‐rank test was used for statistic associations. Continuous and categorical variables were analyzed by t test and *χ* ^2^ test, respectively. *P *< 0.05 was defined as statistical significance.

## Results

### Patient characteristics

Totally, 317 patients were enrolled in our study with a male/female ratio of 2.1 (Table 1). Median age was 61 years (range, 20–92), and 53.3% patients were older than 60 years; 163 patients were newly diagnosed, of whom 67 had treatment indication; 97 patients were previously diagnosed elsewhere, of whom 31 patients had treatment indication. Thirty‐three were relapsed, and 24 were refractory. Median time from diagnosis to sampling was 3 months (range, 0–264).

**Table 1:** Clinical features of patients

Characteristics	*N*
Gender (*n *= 317)	
Male	216
Female	101
Age (*n *= 317)	
>60	169
≤60	148
Rai (*n *= 302)	
0	27
1–2	150
3–4	125
Binet (*n *= 302)	
A	98
B	92
C	112
Disease status (*n *= 317)	
Untreated	260
Newly diagnosis	
With treatment indication	67
No treatment indication	96
Diagnosed elsewhere	
With treatment indication	31
No treatment indication	66
Relapsed	33
Refractory	24

Median follow‐up was 28 months (range, 3–289), during which 215 patients (67.8%) received treatment including rituximab‐based therapy (72/215, 33.5%), chemotherapy alone (112/215, 52.1%), ibrutinib (4/215, 1.9%), and data not available (27/215, 12.5%). Eventually, 70 patients turned to second‐line treatment because of disease progression, and 25 patients received third‐line therapy.

### Sequencing results

Mutation frequencies of *NOTCH1* were 8.2% (26/317) and 0.9% (3/317) in the PEST domain and 3′ UTR, respectively (Table S1). Among patients with PEST domain mutations, 21 cases harbored c.7541_7542delCT, a common frameshift deletion. In addition, c.7443delC, c.7210C>T, c.7378G>T, c.7410delC, and c.7222delC were also detected in the study cohort and all 3′UTR mutations were c.*371A>G. The characteristics of these three patients with 3′UTR mutation are shown in Table S2.

### Clinical and biological characteristics

There were 27 patients in Rai 0 stage (8.9%), 150 in Rai 1–2 (49.7%), and 125 in Rai 3–4 (41.4%). The number of patients with Binet A, B, and C was 98 (32.4%), 92 (30.5%), and 112 (37.1%), respectively. Totally, 78 patients were CD38 positive (27.1%) and 118 were ZAP‐70 positive (46.1%). In addition, 165 patients harbored mutated *IGHV* (55.9%).

*NOTCH1* mutations were more common in patients with Binet C stage (*P *= 0.010) and unmutated *IGHV* gene (*P *< 0.001). In addition, *NOTCH1* mutations were related to CD38 ≥ 30% (*P *= 0.010), with no significant association with ZAP‐70 ≥ 20% (*P *= 0.293, Table 2).

**Table 2:** The relationships between *NOTCH1* mutations and clinical features

Variables	Total (*n*)	*NOTCH1* mutation (*n*)	*NOTCH1* wild type (*n*)	*P* value
Gender	317	29	288	
Male	216	23	193	0.176
Female	101	6	95	
Age	317	29	288	
>60	169	16	153	0.833
≤60	148	13	135	
Rai	302	28	274	
0	27	0	27	0.086
1–2	150	12	138	
3–4	125	16	109	
Binet	302	28	274	
A	98	2	96	0.010
B	92	11	81	
C	112	15	97	
CD38	288	27	261	
≥30%	78	13	65	0.010
<30%	210	14	196	
ZAP‐70	256	23	233	
≥20%	118	13	105	0.293
<20%	138	10	128	
*IGHV* status	295	29	266	
*IGHV* M	165	6	159	<0.001
*IGHV* UM	130	23	107	

### Cytogenetics

Complex karyotype was defined as ≥3 unrelated chromosome abnormalities in more than one cell on karyotype. Totally, 53 patients harbored complex karyotype (19.7%), of whom 7 had *NOTCH1* mutations. The number of patients who harbored del (11) (q22‐23) (11q‐), del (13) (q14) (13q‐), del (17) (p13) (17p‐), and +12 was 39 (15.0%), 39 (13.5%), 79 (38.5%), and 59 (25.4%), respectively. The incidence of 13q‐ was relatively lower than previous study 27, which may due to more Binet C stage patients in our study and the differences of CLL genetic background between Eastern and Western countries patients 26.

The relationship between *NOTCH1* mutations and chromosome abnormalities was shown in Table 3. *NOTCH1* mutations were enriched in CLL patients with +12 (10 of 59 vs. 11 of 173; *P *= 0.014). No significant correlation was found between *NOTCH1* mutations and 11q‐ (*P *= 0.210), 17p‐ (*P *= 1.000), 13q‐ (*P *= 0.053), or complex karyotype (*P *= 0.406).

**Table 3:** Associations between *NOTCH1* mutations and chromosome abnormalities

Variables	Total (*n*)	*NOTCH1* mutation (*n*)	*NOTCH1* wild type (*n*)	*P* value
Complex karyotype	269	25	244	
Positive	53	7	46	0.406
Negative	216	18	198	
11q‐	260	23	237	
Positive	39	6	33	0.210
Negative	221	17	204	
17p‐	288	26	262	
Positive	39	4	35	1.000
Negative	249	22	227	
13q‐	205	21	184	
Positive	79	4	75	0.053
Negative	126	17	109	
+12	232	21	211	
Positive	59	10	49	0.014
Negative	173	11	162	

### CD20 expression intensity

A total of 13 patients with *NOTCH1* mutations had data of CD20 expression intensity, while 138 of *NOTCH1*‐unmutated patients did. We found that *NOTCH1‐*mutated patients had lower CD20 expression intensity than unmutated patients (mean fluorescence intensity (MFI): 614.10 ± 430.71 vs. 951.84 ± 994.39, *P *= 0.029).

In addition, we analyzed the relationship between +12 and CD20 expression intensity. The number of patients who had data of CD20 expression intensity with and without +12 was 29 and 93, respectively. We found that +12 patients had higher CD20 expression intensity than patients without +12 (MFI: 796.90 ± 903.92 vs. 1294.70 ± 1167.18, *P *= 0.018).

Furthermore, we studied the synergetic effect of the status of *NOTCH1* mutations and +12 on CD20 expression. We stratified our patients into four groups: (1) *NOTCH1‐*mutated and +12 (*n *= 4) (*NOTCH1 *M & +12); (2) *NOTCH1‐*unmutated and +12 (*n *= 25) (*NOTCH1* UM & +12); (3) *NOTCH1‐*mutated and +12 negative (*n *= 6) (*NOTCH1 *M & +12 neg); and (4) *NOTCH1‐*unmutated and +12 negative (*n *= 87) (*NOTCH1* UM & +12 neg). *NOTCH1‐*unmutated and +12 patients had higher CD20 expression than those with *NOTCH1‐*mutated and +12 negative (MFI: 1401.36 ± 1219.30 vs. 524.61 ± 265.72, *P *= 0.049) or *NOTCH1‐*unmutated and +12 negative patients (MFI: 1401.36 ± 1219.30 vs. 815.68 ± 929.76, *P *= 0.009) (Tables 4 and 5).

**Table 4:** CD20 expression intensity of different groups stratified according to the status of *NOTCH1* mutations and +12

Groups (*n*)	MFI (mean ± SD)
*NOTCH1 *M & +12 (4)	628.10 ± 365.98
*NOTCH1* UM & +12 (25)	1401.36 ± 1219.30
*NOTCH1 *M & +12 neg (6)	524.61 ± 265.72
*NOTCH1* UM & +12 neg (87)	815.68 ± 929.76

MFI, mean fluorescence intensity; SD, standard deviation; M, mutated; UM, unmutated; neg, negative.

**Table 5:** Hierarchical stratification of CD20 expression intensity according to the status of *NOTCH1* mutations and +12

Pairwise comparisons	*NOTCH1 *M & +12	*NOTCH1* UM & +12	*NOTCH1 *M & +12 neg
*NOTCH1* UM & +12	*P *= 0.141	–	–
*NOTCH1* M & +12 neg	*P *= 0.869	*P *= 0.049	–
*NOTCH1* UM & +12 neg	*P *= 0.706	*P *= 0.009	*P *= 0.478

M, mutated; UM, unmutated; neg, negative.

### Survival

**Figure 1:**
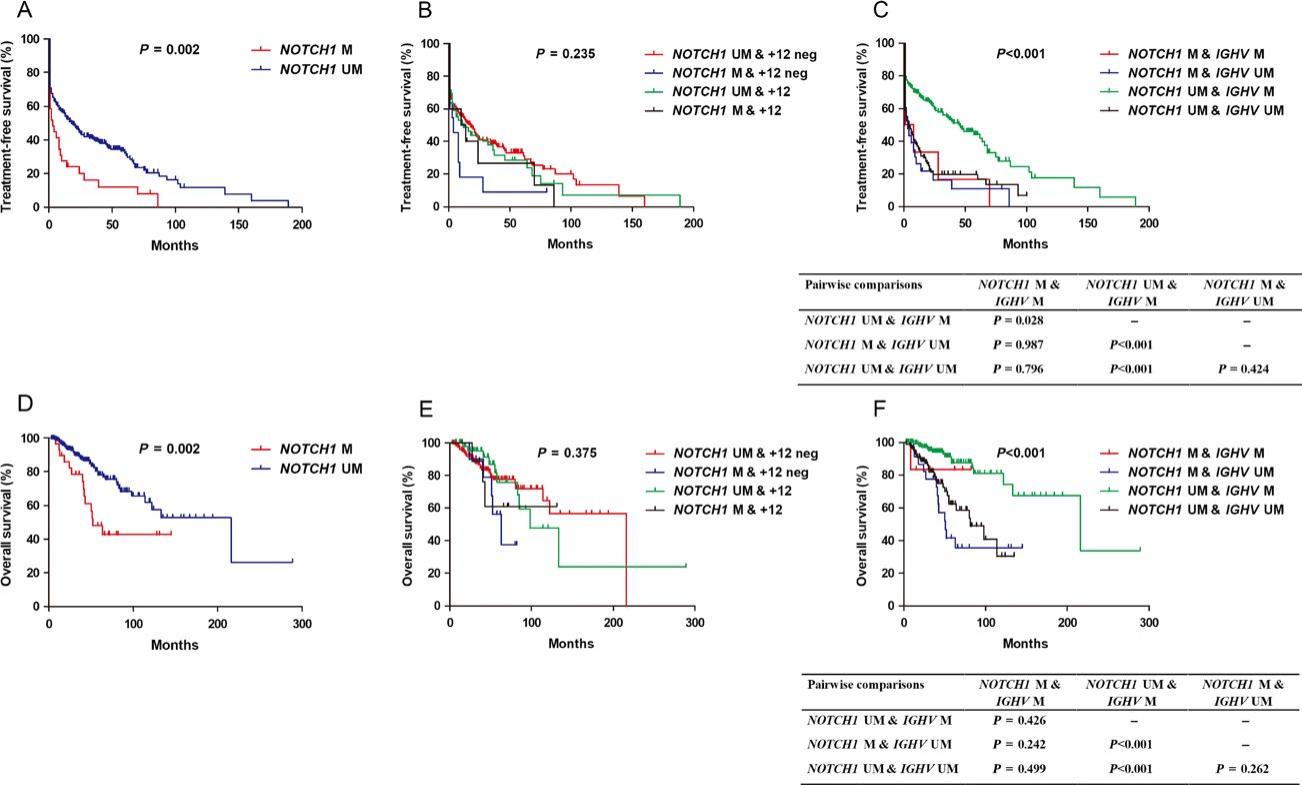
A and D: Kaplan–Meier curves of TFS (A) and OS (D) for *NOTCH1* mutation status. B and E: Hierarchical stratification of TFS (B) and OS (E) according to +12 and *NOTCH1* mutation status. C and F: Hierarchical stratification of TFS (C) and OS (F) according to *IGHV* and *NOTCH1* mutation status. Abbreviations: UM: unmutated; M: mutated; neg: negative.

Patients with *NOTCH1* mutation had worse TFS (Fig. 1A, median: 3 vs. 18 months; *P *= 0.002) and OS (Fig. 1D, median: 52 vs. 216 months; *P *= 0.002) than those with wild‐type *NOTCH1*. Taking the status of *NOTCH1* mutations and +12 into consideration at the same time, we further stratified our patients into four groups as previously mentioned: (1) *NOTCH1 *M & +12 (*n *= 10); (2) *NOTCH1* UM & +12 (*n *= 49); (3) *NOTCH1 *M & +12 neg (*n *= 11); and (4) *NOTCH1* UM & +12 neg (*n *= 162). There was no TFS (*P *= 0.235) and OS (*P *= 0.375) difference among four cohorts (Fig. 1B and E).

The numbers of patients with *NOTCH1‐*mutated and *IGHV‐*mutated (*NOTCH1 *M & *IGHV* M), *NOTCH1‐*unmutated and *IGHV‐*mutated (*NOTCH1* UM & *IGHV* M), *NOTCH1‐*mutated and *IGHV‐*unmutated (*NOTCH1 *M & *IGHV* UM), and *NOTCH1‐*unmutated and *IGHV‐*unmutated (*NOTCH1* UM & *IGHV* UM) were 6, 159, 23, and 107, respectively. Patients with mutated *NOTCH1* and unmutated *IGHV* had worse TFS (Fig. 1C, median: 3 vs. 45 months, *P *< 0.001) and OS (Fig. 1F, median: 51 vs. 216 months, *P *< 0.001) than those with unmutated *NOTCH1* and mutated *IGHV*. In addition, patients with mutated *NOTCH1* and mutated *IGHV* (median: 1 vs. 45 months, *P *= 0.028) or unmutated *NOTCH1* and unmutated *IGHV* (median: 4 vs. 45 months, *P *< 0.001) showed shorter TFS than unmutated *NOTCH1* and mutated *IGHV*. Patients with unmutated *NOTCH1* and unmutated *IGHV* had worse OS than those with unmutated *NOTCH1* and mutated *IGHV* (median: 81 vs. 216 months, *P *< 0.001).

**Figure 2:**
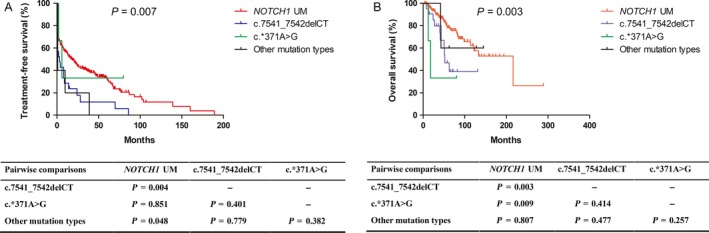
Kaplan–Meier curves of TFS (A) and OS (B) for *NOTCH1* mutation types. Other *NOTCH1* mutation types include c.7443delC, c.7210C>T, c.7378G>T, c.7410delC, and c.7222delC. Abbreviations: UM: unmutated.

It was unknown whether different mutation types had different impacts on prognosis. We allocated patients into four groups: (1) *NOTCH1‐*unmutated (*n *= 288); (2) c.7541_7542delCT (*n *= 21); (3) c.*371A>G (*n *= 3); and (4) other *NOTCH1* mutation types (*n *= 5). *NOTCH1‐*unmutated patients had longer TFS than those with c.7541_7542delCT (median: 18 vs. 3 months, *P *= 0.004) or other mutation types (median: 18 vs. 1 months, *P *= 0.048). Moreover, patients with unmutated *NOTCH1* had longer OS than those with c.7541_7542delCT (median: 216 vs. 52 months, *P *= 0.003) or c.*371A>G (median: 216 vs. 18 months, *P *= 0.009) (Fig. 2).

## Discussion

In the present research, we analyzed clinical characteristics and outcome of 317 Chinese CLL patients with different *NOTCH1* mutational status. The mutation frequencies of PEST domain and noncoding domains were 8.2% and 0.9%, respectively, which were lower than those reported in Western countries 28. Moreover, only c.*371A>G was detected in noncoding domains, with no c.*378A>G or c.*380A>C. Several reasons might account for these different results: first, the differences of CLL genetic background between Eastern and Western countries patients. Second, the frequencies of 3′ UTR mutations were relatively low, with 2.0% for c.*378A>G and 0.8% for c.*380A>C 5. Third, the low sensitivity of Sanger sequencing may contribute. Previous studies have demonstrated that some *NOTCH1* mutations, especially those in noncoding domains, had too low variant frequency to be detected by Sanger sequencing, but could be found by next‐generation sequencing 5. Consequently, mutation frequencies reported by our study might be lower than the true level, and to better study *NOTCH1* mutations, these limitations should be taken into consideration.

The correlation between *NOTCH1* mutations and chromosome abnormalities was assessed in the current study. *NOTCH1* mutations were more common in advanced stage patients and were associated with CD38 positivity and unmutated *IGHV* 22. Complex karyotype, 11q‐, 13q‐, and 17p‐ were not associated with *NOTCH1* mutations. However, patients with *NOTCH1* mutations usually carried +12 21.

The relationships between *NOTCH1* mutations or +12 and CD20 expression intensity were also performed in our study. Patients with *NOTCH1* mutations had lower CD20 expression intensity, and +12 was associated with higher expression intensity of CD20. These findings had clinical value to guide the usage of rituximab for patients who had those two abnormities.

*NOTCH1* mutation represented an unfavorable prognosis factor, which reflected on both TFS and OS 29. By subgroup analysis, no statistic difference for TFS or OS was found among four groups stratified by *NOTCH1* and +12 status. Both *NOTCH1* mutations and unmutated *IGHV* gene had unfavorable effects on survival, but their synergistic effects remained unknown. In our research, we studied survivals of different mutation status of *NOTCH1* and *IGHV*. We found that patients with both mutated *NOTCH1* and unmutated *IGHV* had shorter OS and TFS than those with unmutated *NOTCH1* or mutated *IGHV*. Frameshift deletion c.7541_7542delCT represented unfavorable outcomes for TFS and OS, and c.*371A>G had a negative effect on OS. As a result, CLL patients with c.7541_7542delCT and c.*371A>G mutation deserve more attention in the clinic practice.

In summary, we analyzed clinical and molecular features of *NOTCH1* mutations in 317 Chinese patients with CLL. The frequencies of mutations in the PEST domain and 3′ UTR of *NOTCH1* were lower than those reported by Western countries. *NOTCH1* mutations were more common in patients with advanced stage, unmutated *IGHV* gene, and +12. Moreover, *NOTCH1* mutation represented an unfavorable prognostic factor, especially for c.7541_7542delCT and c.*371A>G. Further researches are needed to better understand the molecular mechanisms of *NOTCH1* mutations and study the appropriate strategies to treat Chinese patients with CLL who harbor *NOTCH1* mutations.

## Conflict of Interest

The authors declare no conflict of interest.

## Supporting information


**Table S1.** *NOTCH1* mutations identified by Sanger sequencing in 317 Chinese CLL cases.
**Table S2.** Clinical characteristics of patients with *NOTCH1* 3′UTR mutation.
