# Supplementary material for: NOTCH1 mutation and its prognostic significance in Chinese chronic lymphocytic leukemia: a retrospective study of 317 cases
Source: Cancer Med. 2018 Mar 23;7(5):1689–96. doi: 10.1002/cam4.1396 (PMC5943423; doi:10.1002/cam4.1396)
Supplement: Supplementary file 1 — Table S1. NOTCH1 mutations identified by Sanger sequencing in 317 Chinese CLL cases. Table S2. Clinical characteristics of patients with NOTCH1 3′UTR mutation. [file CAM4-7-1689-s001.docx]

**Supplementary Material**

Table SI1. *NOTCH1* mutations identified by Sanger sequencing in 317 Chinese CLL cases

| Patient ID | Exon | Nucleotide change | Amino acid change | Refseq |
| --- | --- | --- | --- | --- |
| 6 | 34 | c.7541_7542delCT | p.P2514fs*4 | NM_017617.4 |
| 13 | 34 | c.7541_7542delCT | p.P2514fs*4 | NM_017617.4 |
| 18 | 3’UTR | c.*371A>G | p.? | NM_017617.4 |
| 37 | 3’UTR | c.*371A>G | p.? | NM_017617.4 |
| 49 | 34 | c.7541_7542delCT | p.P2514fs*4 | NM_017617.4 |
| 50 | 34 | c.7541_7542delCT | p.P2514fs*4 | NM_017617.4 |
| 51 | 34 | c.7541_7542delCT | p.P2514fs*4 | NM_017617.4 |
| 55 | 34 | c.7541_7542delCT | p.P2514fs*4 | NM_017617.4 |
| 70 | 34 | c.7443delC | p.L2482fs*1 | NM_017617.4 |
| 95 | 34 | c.7541_7542delCT | p.P2514fs*4 | NM_017617.4 |
| 98 | 34 | c.7210C>T | p.Q2404* | NM_017617.4 |
| 99 | 34 | c.7378G>T | p.E2460* | NM_017617.4 |
| 126 | 34 | c.7541_7542delCT | p.P2514fs*4 | NM_017617.4 |
| 129 | 34 | c.7410delC | p.S2471fs*6 | NM_017617.4 |
| 131 | 34 | c.7541_7542delCT | p.P2514fs*4 | NM_017617.4 |
| 132 | 34 | c.7541_7542delCT | p.P2514fs*4 | NM_017617.4 |
| 174 | 34 | c.7541_7542delCT | p.P2514fs*4 | NM_017617.4 |
| 178 | 34 | c.7541_7542delCT | p.P2514fs*4 | NM_017617.4 |
| 188 | 34 | c.7541_7542delCT | p.P2514fs*4 | NM_017617.4 |
| 197 | 34 | c.7541_7542delCT | p.P2514fs*4 | NM_017617.4 |
| 220 | 34 | c.7541_7542delCT | p.P2514fs*4 | NM_017617.4 |
| 238 | 34 | c.7222delC | p. L2049fs*1 | NM_017617.4 |
| 249 | 34 | c.7541_7542delCT | p.P2514fs*4 | NM_017617.4 |
| 261 | 34 | c.7541_7542delCT | p.P2514fs*4 | NM_017617.4 |
| 285 | 3’UTR | c.*371A>G | p.? | NM_017617.4 |
| 301 | 34 | c.7541_7542delCT | p.P2514fs*4 | NM_017617.4 |
| 308 | 34 | c.7541_7542delCT | p.P2514fs*4 | NM_017617.4 |
| 311 | 34 | c.7541_7542delCT | p.P2514fs*4 | NM_017617.4 |
| 313 | 34 | c.7541_7542delCT | p.P2514fs*4 | NM_017617.4 |

Abbreviations: UTR: untranslated regions.

Table SI2: Clinical characteristics of patients with *NOTCH1* 3’UTR mutation

| Patient ID | Gender | Age | Concomitant alterations | *IGHV* mutational status | CD38 | ZAP-70 | Karyotype | Disease status | TFS(mo) | OS(mo) |
| --- | --- | --- | --- | --- | --- | --- | --- | --- | --- | --- |
| 18 | Male | 77 | *TP53* mutation; del(11q); del(17p); del(13q) | UM | Negative | 49% | 40-42,X,-Y,-1,2p+,-3,3q-,-5,der(7;8)(p10;q10),+der(7),add(11)(p15),-13,17p-,+der(17)add(17)(q25)del(17)(p13),-18,-22,+mar[10cp] | Newly diagnosed | 80+ | 80+ |
| 37 | Male | 65 | NA | UM | Negative | NA | normal | Newly diagnosed | 6 | 18 |
| 285 | Male | 49 | NA | UM | 78% | 28.7% | 43,XY,2q-,6q+,-9,12q+,-13,17p+,-18[1]/46,XY[19] | Refractory | 2 | 12 |

Abbreviations: UM: unmutated; M: mutated; NA: not available; mo: month; *IGHV*: immunoglobulin heavy chain variable region; UTR: untranslated regions.
